# Surface-Modified and Unmodified Calcite: Effects of Water and Saturated Aqueous Octanoic Acid Droplets on Stability and Saturated Fatty Acid Layer Organization

**DOI:** 10.1021/acs.langmuir.1c02387

**Published:** 2021-11-18

**Authors:** Natalia A. Wojas, Agne Swerin, Viveca Wallqvist, Mikael Järn, Joachim Schoelkopf, Patrick A. C. Gane, Per M. Claesson

**Affiliations:** † Division of Bioeconomy and Health, Materials and Surface Design Department, RISE Research Institutes of Sweden, Box 5607, SE 114 86 Stockholm, Sweden; ‡ School of Engineering Sciences in Chemistry, Biotechnology and Health, Department of Chemistry, Division of Surface and Corrosion Science, KTH Royal Institute of Technology, Drottning Kristinas väg 51, SE-100 44 Stockholm, Sweden; § Faculty of Health, Science and Technology, Department of Engineering and Chemical Sciences: Chemical Engineering, Karlstad University, SE-651 88 Karlstad, Sweden; ∥ Omya International AG, Baslerstrasse 42, CH-4665 Oftringen, Switzerland; ⊥ School of Chemical Engineering, Department of Bioproducts and Biosystems, Aalto University, P.O. Box 16300, FI-00076 Aalto, Finland

## Abstract

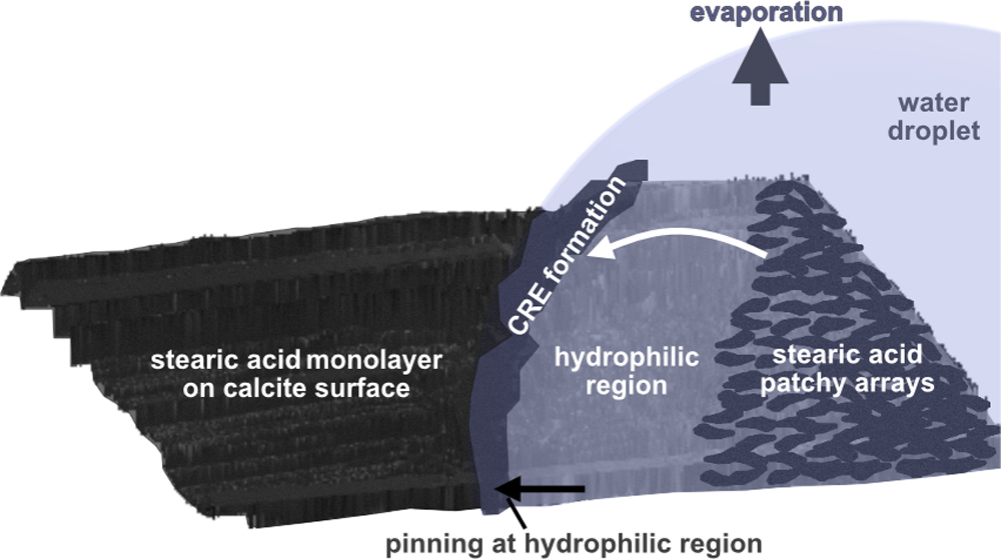

A profound understanding of the properties of unmodified and saturated fatty acid-modified calcite surfaces is essential for elucidating their resistance and stability in the presence of water droplets. Additional insights can be obtained by also studying the effects of carboxylic acid-saturated aqueous solutions. We elucidate surface wettability, structure, and nanomechanical properties beneath and at the edge of a deposited droplet after its evaporation. When calcite was coated by a highly packed monolayer of stearic acid, a hydrophilic region was found at the three-phase contact line. In atomic force microscopy mapping, this region is characterized by low adhesion and a topographical hillock. The surface that previously was covered by the droplet demonstrated a patchy structure of about 6 nm height, implying stearic acid reorganization into a patchy bilayer-like structure. Our data suggest that during droplet reverse dispensing and droplet evaporation, pinning of the three-phase contact line leads to the transport of dissolved fatty carboxylic acid and possibly calcium bicarbonate Ca(HCO_3_)_2_ molecules to the contact line boundary. Compared to the surface of intrinsically hydrophobic materials, such as polystyrene, the changes in contact angle and base diameter during droplet evaporation on stearic acid-modified calcite are strikingly different. This difference is due to stearic acid reorganization on the surface and transport to the water–air interface of the droplet. An effect of the evaporating droplet is also observed on unmodified calcite due to dissolution and recrystallization of the calcite surface in the presence of water. In the case where a water droplet saturated with octanoic acid is used instead of water, the stearic acid-coated calcite remains considerably more stable. Our findings are discussed in terms of the coffee-ring effect.

## 1 Introduction

Calcium carbonate (CaCO_3_) is the most abundant inorganic biomineral in nature, which is due to the predominance of limestone over other carbonate rocks.^1^ Calcium carbonate is a polymorphous, variform compound, which nucleates into three different crystal modifications: calcite, aragonite, and vaterite.^2−4^ Most limestone sources consist essentially of calcite as it is the thermodynamically most stable polymorph under ambient conditions.^1^ The most common crystal system of calcite is the rhombohedral one, which exposes {101̅4} faces, and is frequently used in experimental and theoretical studies as a model structure.^2,5^ With all their advantages, calcium carbonate rock, that is, limestone, marble, and chalk, enjoy considerable industrial utilization.^2,6,7^ They are important in the pharmaceutical, paper, plastic, and food industries and are also used in water purification as precipitates strengthening the soils and as a sorbent for exhaust gasses.^2,8−13^ Moreover, it is considered as a key mineral to build many organisms’ exoskeletons for protecting and supporting purposes, as well as tissues for light perception and storage of calcium ions.^2,5,14,15^ Understanding the surface chemistry of calcium carbonates and their interactions with other substances is thus essential. In particular, the dynamic nature of the calcite surface is an important issue where much information can be gained by use of scanning probe methods.^16−18^

In many industrial applications, CaCO_3_ particles act as fillers embedded in an organic matrix. To achieve the desired product properties, the synergy between the filler and matrix needs to be optimized. Thus, it is essential to understand the interactions occurring at organic–inorganic interfaces. Different classes of compounds have been evaluated as surface modification agents for calcite, and carboxylic acids are commonly used for this purpose.^19−26^ The high surface energy and hydrophilic surface of CaCO_3_ are incompatible with a low-energy surface of, for example, a non-polar polymer matrix. Therefore, surface treatment of calcite is needed. It is often aimed to improve not only the adhesion and dispersibility but also the mechanical properties, including tensile strength, stiffness and elongation, abrasion resistance, viscosity, and so forth.^27−30^ In order to achieve full benefits from modified mineral fillers, especially for moisture curing applications, it is essential to minimize pickup of undesired organic molecules and water. A water film initiates undesired generation of hydrated calcium bicarbonate that alters the adsorption of modifiers.^31,32^ Calcium carbonate has limited solubility (14 mg/L) in pure water.^2,33^ However, if carbon dioxide is present, the solubility increases by more than a factor of 100, and the carbonate ion goes into solution as a hydrogen carbonate ion^2^
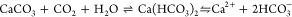
1

As calcium carbonate has a high surface energy (about 500–600 mJ/m^2^ for the {101̅4} plane), it also readily adsorbs organic molecules.^34,35^ Therefore, for fundamental studies, a clean, freshly fractured CaCO_3_ surface should be used for effective surface modification, after which the modified surface should be stored in the dry pure air (or preferably vacuum) environment. This is, however, not realistically achievable in industrial processes.^36^ The calcite surface itself is highly reactive and readily undergoes recrystallization with time in air and particularly at high relative humidity. When the calcite surface is modified with fatty carboxylic acids, the adsorption and layer reorganization will compete and combine with hydration, dissolution, and recrystallization,^6,17^ especially under mechanical wear and humid conditions.^18^

For a profound understanding of the properties of fatty carboxylic acid-modified calcite surfaces, it is essential to elucidate their resistance to water droplets, both below the droplet and at the droplet edge. In this work, we followed the evolution of the surface wettability, structure, and nanomechanical properties of pure and stearic acid-modified calcite surfaces when exposed to deposited droplets of pure water and water saturated with octanoic acid. Our results highlight the importance of desorption into the bulk, and at the edge of, the droplet and layer reorganization. As a result, the hydrophobized calcite surface shows strikingly different wetting properties compared to the surfaces of intrinsically hydrophobic materials. We utilize atomic force microscopy (AFM) topographical and nanomechanical imaging to elucidate the effects of the water droplet on bare calcite and carboxylic acid-modified calcite surfaces. Our data show that the presence of aqueous droplets leads to uneven adsorption due to layer rearrangements, dissolution, and capillary flow. Our results are discussed in light of the coffee-ring effect (CRE),^37−40^ where capillary flow transports non-volatile material to the edge of the droplet. Experiments with octanoic acid-saturated aqueous droplets show that the CRE can be suppressed to achieve higher stability of the initially homogeneous fatty acid layer.

## 2 Experimental Section

### 2.1 Materials

The material used in the experiments was of optical quality Iceland spar calcite (mined in Madagascar and purchased from Geocity AB, Stockholm). A rhombohedral crystal was fractured into smaller samples with a stainless steel chisel and hammer^17^ along the dominant {101̅4} cleavage plane. This highly hydrophilic surface^34,41^ was immediately purged with pressurized nitrogen (nitrogen ≥99.9 vol %, oxygen ≤20 ppm, water ≤10 ppm) to remove debris and minimize adsorption of airborne molecules. At least three samples (for each study) of about 3 mm thick, and with surface areas in the range of 25–450 mm^2^ exhibiting no evidence of excessive microcracks and steps, were chosen for the measurements. An epoxy glue (Bostik) was utilized for the sample attachment to the magnetic disc used in AFM experiments. The following saturated carboxylic acids were utilized for the modification of the calcite surface: octanoic acid (C_8_, known as caprylic acid) for synthesis ≥99.0% (Sigma-Aldrich) and octadecanoic acid (C_18_, known as stearic acid) for synthesis ≥97.0% (Sigma-Aldrich). Polystyrene, in the form of a Petri dish, was used as a reference sample for studies concerning intrinsically hydrophobic materials. Silica gel granulate (Merck) was used in order to maintain low humidity conditions (below 2.5 %RH) during carboxylic acid vapor exposure at room temperature. The relative humidity was measured with an external sensor (HMT317, Vaisala), placed in close proximity to the samples. Ultrapure Milli-Q water (type 1, ASTM D1193-91) was utilized as water medium in all studies. The laboratory room temperature was set at 23 ± 0.5 °C, and the room relative humidity varied in the range 25–35%.

### 2.2 Calcite Surface Modification with Carboxylic Acids

The CaCO_3_ surface was modified by exposure to carboxylic acid vapor. Surface modification via carboxylic acid vapor exposure is, however, efficient only at high enough vapor pressure and thus strongly dependent on the surrounding temperature.^42^ For this reason, modification has to be performed above the melting temperature. The vapor pressures of the fatty acids under the deposition conditions are presented in Table 1.

**Table 1:** Melting Point and Vapor Pressure at the Deposition Temperature and Aqueous Solubility of Octanoic and Stearic Acid^33,42^

Acid	Melting point (°C)	Vapor pressure (Pa) at deposition temperature	Aqueous solubility (g/kg) at 25 °C
C_8_	16.5	0.49^liq^ at 25 °C	0.800
C_18_	69.3	355^liq^ at 105 °C	0.003

For modification of the calcite surface with octanoic acid, the samples were stored inside a closed desiccator (volume about 2.3 L) for 4 h at room temperature (about 23 °C). The desiccator contained 40–50 mL of octanoic acid in a flat glass beaker (diameter about 57 mm). Another beaker with silica granulates was placed below the porcelain plate to stabilize the low humidity inside the desiccator at room temperature, so that calcite recrystallization was retarded.^17^

For modification by stearic acid, an oven (UF 55, Memmert) was used and set for 4 h at a temperature of 105 °C, and the relative humidity was estimated to be below 2.5 %RH. The temperature and time were chosen to achieve the full monolayer coverage as judged by the high initial water contact angle (CA). The samples were kept in an unsealed 1.5 L glass box containing a glass beaker filled with 20–25 g of stearic acid that was previously melted at the same temperature for about 90 min.

### 2.3 Saturated Octanoic Acid Solution

To study surface interactions with water, one should not only consider pure water but also saturated solutions of the carboxylic acids used for modification of the calcite surface. Due to the low solubility of stearic acid (Table 1), we have chosen to investigate only solutions saturated with octanoic acid. The C_8_-saturated solution was made by overnight magnetic stirring of octanoic acid with Milli-Q water followed by sedimentation of the heavier C_8_-saturated aqueous solution and decantation of this liquid through separatory funnel drains. The pH of this solution was initially around 4 and found to increase slowly with time in the presence of calcite (see Supporting Information, Figure S1).

The surface tension of octanoic acid and the initial liquids was measured using two methods for accuracy. In the pendant drop shape analysis method, the shape of the drop hanging from a needle is determined from the balance of forces which include gravitational pull and the surface tension of the liquid being investigated. The droplet size was increased by 1 μL/s, and the droplet was released from the needle when the volume reached 20–30 μL.

The second method utilized a platinum Wilhelmy plate and a Force Tensiometer-K100 (Krüss, Germany), where the force acting on a vertically immersed plate is measured. The plate was removed from the liquid at a speed of 10 mm/min, using an initial immersion depth of 2 mm. The measurements were repeated about 20 times. The average values for the surface tension from all measurements and density of the studied solutions are reported in Table 2.

**Table 2:** Average Surface Tension and Density of Milli-Q Water, C_8_-Saturated Milli-Q Water, and Octanoic Acid at 22 °C^a^

Solution	Surface tension(mN/m)	Density(kg/m^3^)^33^
Milli-Q water	71.9 ± 0.4	0.997
C_8_-saturated Milli-Q water	31.5 ± 0.2	-
Octanoic acid (liq)	28.3 ± 0.1	0.911

a The density of C_8_-saturated Milli-Q water has not been measured.

### 2.4 Surface Imaging and Nanomechanical Properties

The morphology and nanomechanical properties of carboxylic acid-modified calcite surfaces exposed to liquid droplets were recorded by utilizing a MultiMode 8 AFM (Bruker) with a standard holder and scanner (S/N: 10578JVLR, Bruker). Special wear-resistant HQ:NSC35/Hard/Al BS probes (MikroMasch) with a nominal tip diameter of <20 nm, resonance frequency of 330 kHz, and nominal spring constant, *k*_*z*_, of 21 N/m were used as they are highly resistant to wear. The actual value of *k*_*z*_ was determined using the thermal tune method within the Nanoscope program.^43^ The deflection sensitivity in the normal direction was found to be 24.2 nm/V with the use of a sapphire calibration sample (Bruker) in air. The modified calcite surfaces were studied immediately after preparation. After locating a relatively smooth area of the sample with a white-light microscope, the edge of a deposited water droplet was found by utilizing a microscope equipped with a camera, also under white light illumination. After removing the water droplet, the edge region and the region previously below the droplet were investigated by AFM. For these studies, the peak force quantitative nanoscale mechanical characterization (QNM) mode was used with the selected applied force of 20 nN. This force was utilized since it deformed the surface sufficiently to allow measurements of nanomechanical properties and yet was sufficiently low to not damage the tip itself, as judged by imaging rough surfaces before and after measurements. Nanomechanical properties were determined using 512 × 512 pixel images over a scanned area from 2 × 2 to 40 × 40 μm^2^ with a scanning frequency of 0.5–1.0 Hz. In all cases, no image enhancement was performed apart from plane fit and flattening of height (second order) images in the NanoScope Analysis program. We note that the exact values of nanomechanical properties, such as adhesion and deformation, in general depend on probe radius and applied force. For this reason, we focus on variations in these properties observed in images taken by the same probe using the same applied force, which remains consistently the case when considering individual nanomechanical images.

### 2.5 Contact Angle

The interactions between modified calcite surfaces and liquids (Milli-Q water, saturated aqueous octanoic acid solution) were measured using an optical CA device (OCA40, Data Physics Instruments GmbH) equipped with an automated micro-pipette. The droplet shape was recorded at the rate of 6 frames/s by a high-resolution CCD camera and analyzed utilizing the ellipse fitting method in the SCA20 software (DataPhysics Instruments GmbH). The average of the CAs on the left and the right side of the droplet was calculated. All CA measurements were carried out immediately after surface modification. At least five samples were analyzed, where each could fit 2–4 independent 1 μL water droplets.

The contact angle hysteresis (CAH) was determined by measuring the advancing and receding CAs. The advancing CA was determined when the droplet volume was increased to 5 μL, after which the droplet was kept on the sample surface for about 30 s. Next, the receding CA was determined when the droplet volume was decreased again. In all these measurements, the speed of the droplet change was set to 1 μL/s. Between measurements, the syringe was cleaned from the concealed liquid by dispensing several droplets (about 15–20 μL) outside the sample surface.

## 3 Results and Discussion

In this section, we first consider morphological and nanomechanical changes that occur on calcite and carboxylic acid-modified calcite surfaces, as a result of exposure to droplets of pure water and water saturated with octanoic acid. Next, we consider the wetting characteristics of carboxylic acid-modified calcite, including CAH and changes in CAs and base diameter (BD) during droplet evaporation.

## 4 Morphological and Nanomechanical Changes

### 4.1 Droplets on Unmodified Calcite

#### 4.1.1 Pure Water

**Figure 1:**
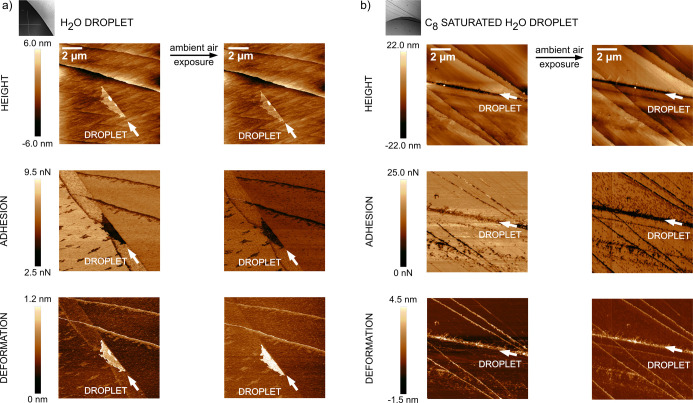
AFM topography and nanomechanical images of the calcite surface after contact with a water droplet (a) and C_8_-saturated water droplet (b), prior and after exposure to 25–35 %RH air overnight. Microscopic images illustrate the position of the droplet. The edge of the aqueous droplet is marked by an arrow. Large features (surface steps) seen in the topography image are due to the crystal structure and not a result of the droplet edge. 40 × 40 μm^2^ images are provided in Figure S2 in Supporting Information.

To gain a clear understanding of how the droplet affects the surface, the morphological and nanomechanical changes were first elucidated. Figure 1a shows the topography, tip-sample adhesion, and surface deformation of a calcite surface area. Here, the lower left part has been covered by a microliter range water droplet for 30 s, while the upper right part has been in contact with air. The border between these two areas is most clearly seen in the adhesion image, and the boarder is located at exactly the same position in the other images as they were collected at exactly the same time and at exactly the same spot on the surface. The calcite surface area that was located under the water droplet and at the edge of the water droplet is different from the area only exposed to air. This is evidently seen in the adhesion and deformation images but less clearly in the topography image. In the adhesion and deformation images, one can distinguish the position of the droplet edge and non-spherical deposits with decreased adhesion and increased deformation under the previously dispensed droplet. The observed changes are due to more extensive dissolution and recrystallization of the calcite surface in contact with water. As discussed in our previous work,^17^ these features are signs of formation of a hydrated form of calcium carbonate, and apparently this transformation occurs more rapidly below the droplet than outside the region previously covered by the droplet. The solubility of calcite in pure water is 14 mg/L,^33^ but it increases significantly in the presence of carbon dioxide due to the formation of calcium bicarbonate Ca(HCO_3_)_2_ (solubility 166 g/L^33^). During prolonged exposure to ambient air, further morphological changes occur also in the areas not exposed to water, not clearly shown here but reported in detail in our previous work.^17^ This is due to the hydrophilic nature of calcite that allows formation of a thin adsorbed water layer on the surface, and we have previously shown that the rate of change increases with increasing relative humidity.^17^

#### 4.1.2 Octanoic Acid-Saturated Water

As shown in Figure 1b, the surface area exposed to the octanoic acid-saturated water droplet is at the lower part of the images. Here, the curved line shown in all images represents the position of the droplet edge. By comparing Figure 1a,b, it is clear that the surface below the droplet is less affected when C_8_-saturated water is added compared to when pure water droplets are used. In particular, we observe a more homogeneous surface and no deposits at the droplet edge, which implies that the calcite surface is partly protected by C_8_ adsorption. Indeed, water CA measurements on a calcite surface dipped into saturated octanoic acid solution show an initial CA of 99° after removal from the solution, demonstrating the formation of an octanoic acid monolayer. Interestingly, we find no nanomechanical contrast between droplet-exposed and unexposed areas. Recrystallization with growing patches on the terraces and steps becomes evident in the adhesion image after overnight air exposure. As before, the tip-surface adhesion is decreased in the recrystallized areas.

### 4.2 Droplets on Octanoic Acid-Modified Calcite

#### 4.2.1 Pure Water

**Figure 2:**
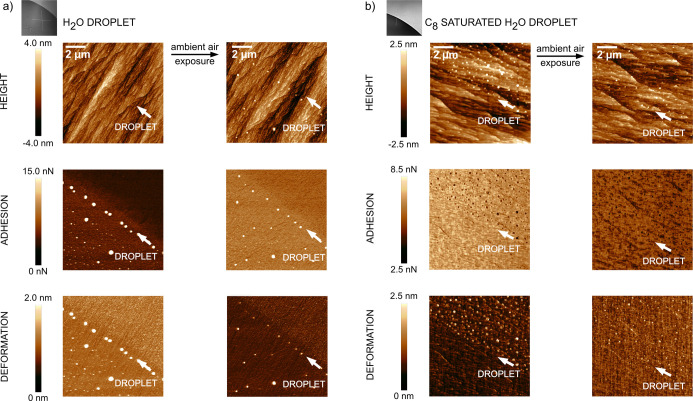
AFM topography and nanomechanical images of the C_8_-modified calcite surface after contact with a water (a) and C_8_-saturated water droplet (b), prior and after exposure to 25–35 %RH air overnight. Microscopic images illustrate the position of the droplet. The edge of the aqueous droplet is marked by an arrow. 40 × 40 μm^2^ images are illustrated in Figure S3 in Supporting Information.

Data obtained for calcite modified by exposure to octanoic acid vapor and then exposed to a water droplet for 30 s are presented in Figure 2a. Here, the droplet covered the lower left part in the images, and the droplet edge is seen clearly in adhesion and deformation images. The border is distinguished by a curved line of well-defined spherical deposits. However, the roughness of the surface does not allow these features to appear evidently in the topography image. They are apparently due to octanoic acid that partly dissolves in the water droplet and accumulates at the air–water interface before being partly deposited again as the water droplet is removed. It is not clear if the deposits only contain octanoic acid or if also dissolved and recrystallized calcium carbonate is included. However, these deposits can be easily smeared out by scanning the AFM tip in the contact mode over the surface (data not shown), even though they remain for hours (images on the right are after 18 h exposure to air) if left untouched in air. Compared to the bare calcite surface, Figure 1a, octanoic acid deposition reduces the recrystallization of the surface upon water exposure. However, due to the C_8_ solubility in water, a structural change of the surface is still observed, but it is now mainly due to the rearrangement of the adsorbed octanoic acid. Thus, our hypothesis is that the solubilization of octanoic acid and redeposition on the surface during droplet removal are the cause for the structural changes, as observed in Figure 2a.

#### 4.2.2 Octanoic Acid-Saturated Water

In case the mentioned hypothesis is correct, no similar changes should be observed if the (partial) solubilization of the adsorbed octanoic acid was prevented. This situation can be achieved using a saturated aqueous C_8_ solution instead of pure water. In this case, exchange of octanoic acid in solution and on the surface can occur, but the overall octanoic acid concentration in the droplet cannot be increased. The data in Figure 2b report on this situation. One can still clearly distinguish the droplet edge in the adhesion and deformation images, but the surface area exposed to the saturated aqueous octanoic acid solution looks homogeneous but with higher adhesion and lower deformation than the unexposed area, suggesting additional adsorption of octanoic acid. In contrast to the case with a water droplet, no spherical deposits are observed at the droplet edge. Apparently, the deposited octanoic acid monolayer is more stable in contact with a saturated octanoic acid solution than that in contact with pure water, which would be expected if solubilization of octanoic acid was the main reason for the structural changes, as observed in Figure 2a. With increasing time in air, the difference in nanomechanical properties between the exposed and unexposed area decreases, suggesting surface diffusion aided by adsorbed water vapor that evens out the initial packing density contrast. We note that in Figure 2b, we see small surface features on the air side. They are distinguished by lower adhesion than the surrounding, which distinguishes them from the C_8_ deposits found on the area covered by the droplet, as shown in Figure 2a. The features, as seen in Figure 2b, are likely debris from the cleavage in the form of hydrated calcite, which is dissolved and/or removed when exposed to the droplet and during droplet removal.

### 4.3 Droplets on Stearic Acid-Modified Calcite

#### 4.3.1 Pure Water

**Figure 3:**
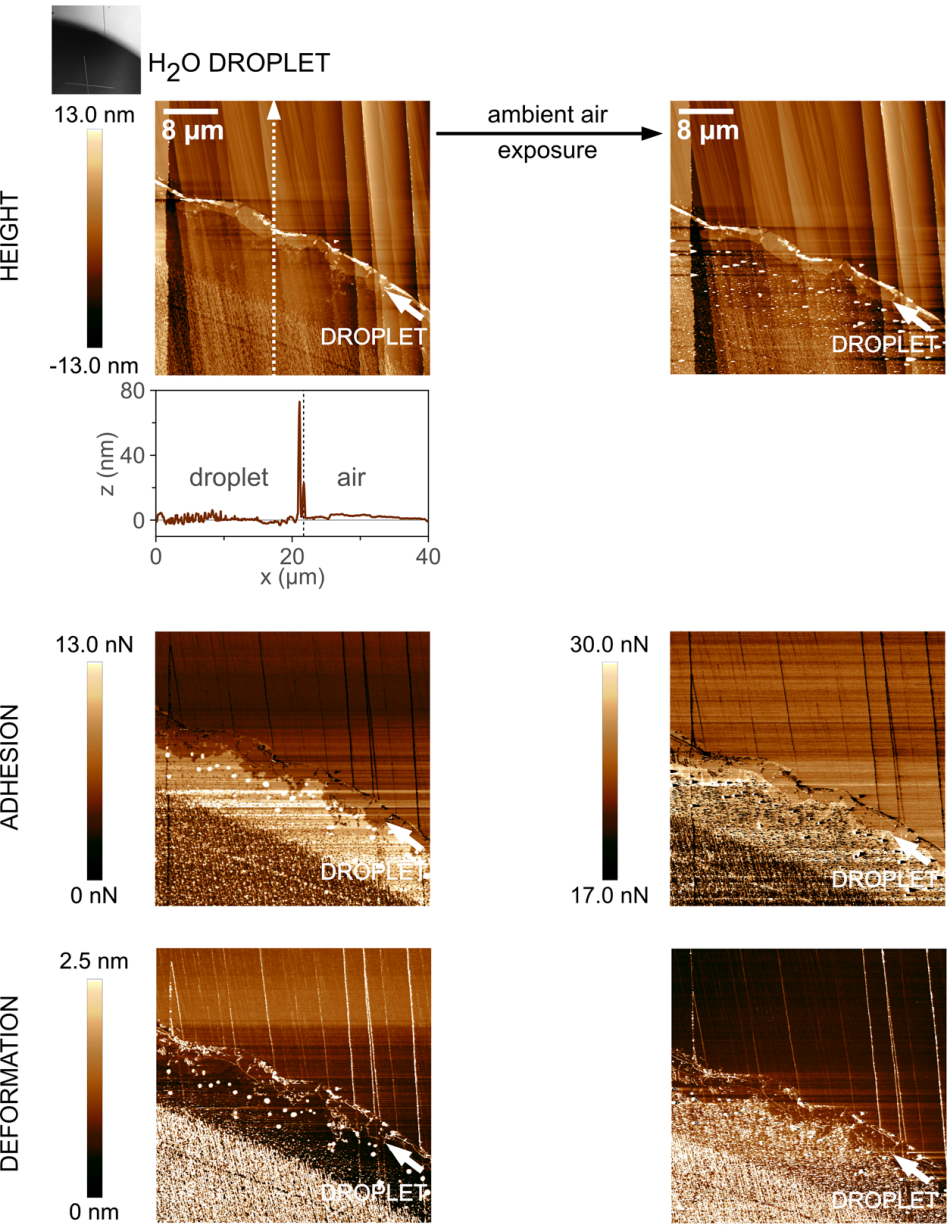
AFM topography and nanomechanical images of the C_18_-modified calcite surface after contact with a water droplet prior and after exposure to 25–35 %RH air overnight. Microscopic images illustrate the position of the droplet. The edge of the aqueous droplet is marked by an arrow. Note the large accumulation of the material at the droplet edge, which is due to the CRE.

**Figure 4:**
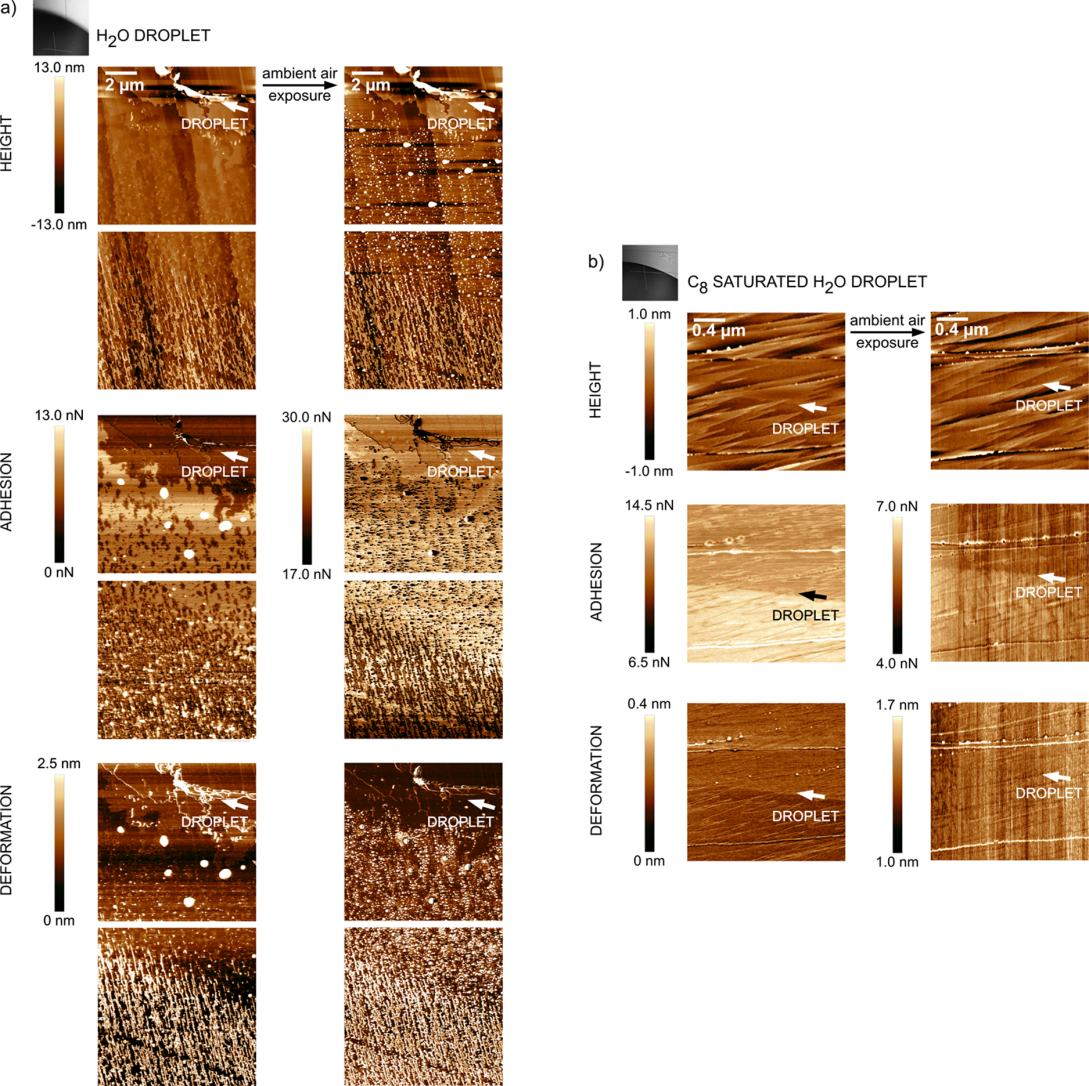
AFM topography and nanomechanical images of the C_18_-modified calcite surface after contact with a water (a) and a C_8_-saturated water droplet (b) prior and after exposure to 25–35 %RH air overnight. Microscopic images illustrate the position of the droplet. The edge of the aqueous droplet is marked by an arrow. The two images with scale bar 2 μm in (a) are focused on areas at the droplet edge (upper one) and below the droplet (lower one). Corresponding 5 × 5 μm^2^ images to those in panel (b) are reported in Figure S4 in Supporting Information. Note the different scale bars in Figure 4a,b.

Stearic acid is much less soluble in water than octanoic acid (Table 1). Thus, one would not expect that dissolution of the C_18_ layer would be of equal importance as for C_8_ layers. However, rearrangements of the layer can still occur. Calcite modified with stearic acid vapor has a very hydrophobic nature (Table 3), resulting from the hydrocarbon chains being exposed outward, while the carboxylic acid groups are attached to the calcite surface. From surface energy considerations, this remains a favorable situation when the surface is in contact with air but not when in contact with water. Thus, one could expect that surface energy minimization would drive a reorientation of the pre-adsorbed stearic acid layer when exposed to a water droplet. This indeed occurs and is shown in Figures 3 and 4a where the water droplet covered the lower left part of the image of the C_18_-modified calcite. The thin irregular line, as seen in the topography image in Figure 3, is due to stearic acid deposition at the pinned contact line. Just below this line follows an area with increased adhesion and decreased deformation that suggests depletion of the stearic acid layer on the water side of the applied droplet edge. Further inside the droplet, the initially homogeneous adsorbed acid layer has been converted into a patchy array over the calcite surface. The height of the patches seen in the topography images in the region exposed to the droplet is about 4.4–6.7 nm, which indeed suggests transformation of the initial monolayer into at least bilayer patches. In comparison, the extended length of the stearic acid molecule is 2.4–2.6 nm.^20,22^

**Table 3:** Initial Contact Angle of Milli-Q Water and C_8_-Saturated Milli-Q Water Droplets on Calcite and Carboxylic Acid-Modified Calcite

Solution	Freshly fractured CaCO_3_	C_8_-modified CaCO_3_	C_18_-modified CaCO_3_
Milli-Q water	<5°	105° ± 3°	108° ± 5°
C_8_-saturated Milli-Q water	70° ± 5°	68° ± 2°	69° ± 2°

A more pronounced accumulation of material at the droplet edge occurs for stearic acid-modified calcite, as observed in Figure 3, compared to the C_8_-modified surface, as illustrated in Figure 2a, which is related to the CRE, discussed later, and the solubility of the carboxylic acids. Octanoic acid can be partly solubilized in the water droplet, whereas C_18_ hardly dissolves but can be transported to the edge via the air–water interface. Compared to the C_8_ case, the border between the exposed and unexposed areas is less regular, which is due to more severe droplet pinning (Figure 3). This, in turn, is due to a larger difference in hydrophobicity between the area under the droplet and at the edge where stearic acid is removed to the air–water interface. The droplet edge region remains clearly seen also after 15–16 h, suggesting high stability of the stearic acid layer in air, and we also note recrystallization of calcite in exposed bare calcite areas (close to the droplet edge), most evidently seen in the nanomechanical images of Figures 3 and 4a.

We note that the images, as shown in Figure 3, cover a significantly larger area than those reported in Figures 1 and 2. The reason for this is that the surface area affected by the water droplet is so large for stearic acid-modified calcite that it cannot be captured fully at higher resolutions. Nevertheless, higher resolution images of the droplet edge region for stearic acid-modified calcite are provided in Figure 4a to facilitate direct comparison with the images reported in Figures 1 and 2.

#### 4.3.2 Octanoic Acid-Saturated Water

When a droplet of C_8_-saturated aqueous solution is used (Figure 4b), a much smaller effect on the surface morphology and nanomechanical properties is observed than with pure water. In fact, in order to see clearly any effect at all, we needed to image small surface areas (note the scale bar in Figure 4b), and here slight differences in adhesion and deformation of the area exposed and not exposed to the droplet can be distinguished. The reason for this is that there is now another mechanism than C_18_ reorientation that can reduce the surface energy between the modified calcite surface and the aqueous solution. This is adsorption of octanoic acid on top of the C_18_ layer, which has the energetic advantage that the strong bond between calcite and the carboxylic acid group is retained, while the interfacial energy between the surface and the aqueous phase is reduced. As a result of predominance of octanoic acid adsorption hardly any contrast or spherical deposits are seen in any of the images in Figure 4b. This also suggests that the outer layer of octanoic acid adsorbed on top of the stearic acid layer by hydrophobic interactions is removed together with the water droplet. By comparing the data reported in Figure 4a,b it is clear that our results demonstrate that the presence of C_8_ in the aqueous solution stabilizes the C_18_ layer significantly.

### 4.4 Surface Wettability Hysteresis

#### 4.4.1 Pure Water

The structural changes observed under the deposited droplets and at the edge of the droplet should also be evident in the wetting behavior. Thus, to gain further understanding, experiments focused on water CAH were performed. Here, the three-phase contact line was advanced during the first 5 s by increasing the droplet volume, then kept at rest for 30 s, and finally withdrawn by reducing the droplet volume. Thus, the surface was in contact with the droplet under the same conditions as analyzed after droplet removal in the previous sections. The initial CAs measured at 1.2 s after droplet deposition for all mentioned samples are stated in Table 3.

**Figure 5:**
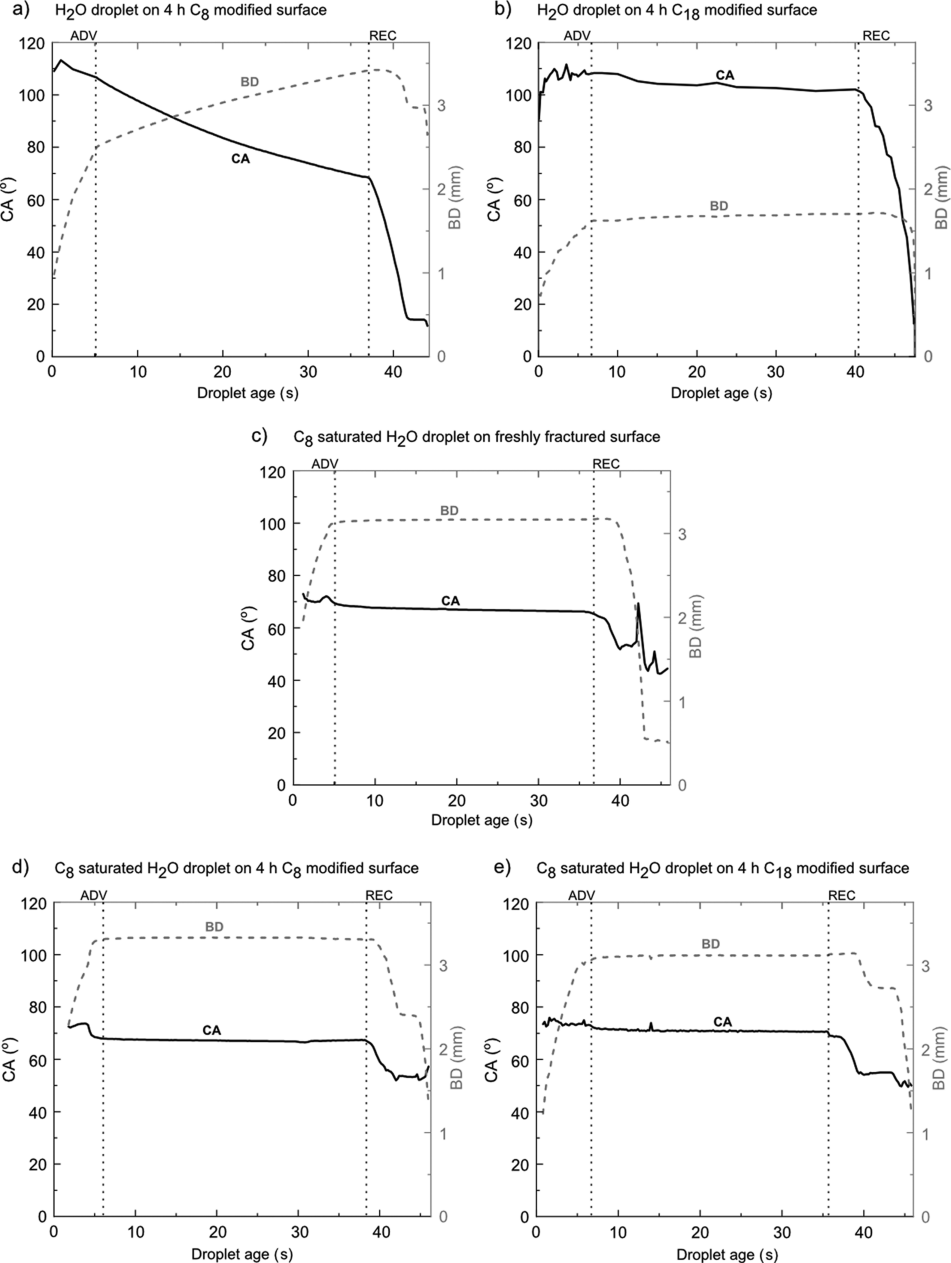
CAH and normalized droplet BD of water droplet on (a) octanoic acid-modified, (b) stearic acid-modified calcite surfaces; and octanoic acid-saturated water droplet on (c) freshly fractured calcite surface, (d) octanoic acid-modified, (e) and stearic acid-modified calcite surfaces.

Droplets of Milli-Q water spread on freshly cleaved calcite, whereas C_8_- and C_18_-modified surfaces are initially hydrophobic with CAs close to 110°. However, with time, the carboxylic acid layer under the droplet and at the droplet edge changes, as demonstrated previously in Figures 2–4. As illustrated here in Figure 5, this results in a decrease in the CA and increase in droplet BD with time. The changes occur much more rapidly for the more soluble C_8_ compared to the less soluble C_18_. Quantitatively, the droplet BD increases by 35% on octanoic acid-modified calcite and by 5% on stearic acid-modified calcite. The water droplet is initially pinned when retracted, which is due to the hydrophilic region created at the droplet edge.

#### 4.4.2 Octanoic Acid-Saturated Water

The CA measured with a saturated aqueous C_8_ solution is in all cases initially around 70°. This means that both for bare calcite and carboxylic acid-modified calcite, we end up with a similar situation, suggesting a monolayer coverage just outside the droplet edge and a full or partial bilayer exposing carboxylic acid groups toward the solution under the droplet due to C_8_ adsorption. When the octanoic acid-saturated droplet is at rest, the CA and BD are much more stable as compared to the case of pure water droplets. This is due to the higher stability of the initial carboxylic acid layer, as demonstrated in Figures 2–4. Quantitatively, we find that the BD increased by less than 2% of the initial values for C_8_-saturated droplets. As the droplet volume was reduced, the BD decreased more readily, that is, in a shorter time after starting the droplet volume reduction process, compared to when a pure water droplet was used. This correlates with the smaller edge effects seen in the AFM images, Figures 2–4. However, the data suggest some pinning also when aqueous-saturated octanoic acid solutions are used.

### 4.5 Wettability of Drying Droplets

#### 4.5.1 Pure Water

**Figure 6:**
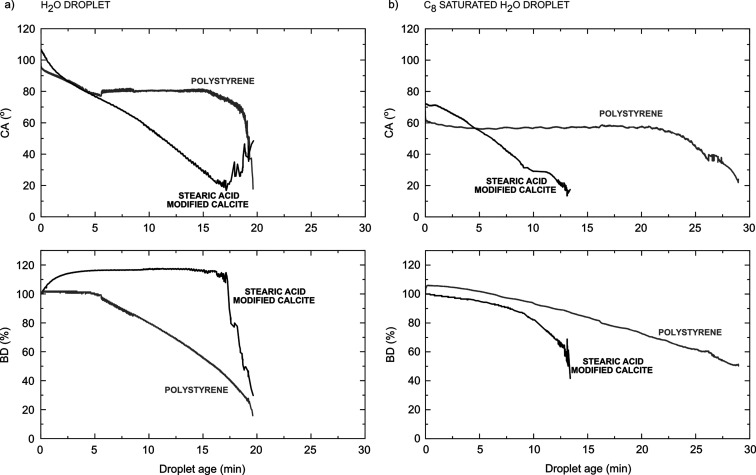
CA and normalized BD as a function of time on stearic acid-modified calcite and polystyrene. Measurements were carried out with (a) water and (b) octanoic acid-saturated water droplets.

In our discussion above, we have emphasized dynamic changes in the adsorbed carboxylic acid layer to rationalize the wetting behavior and found support for this in the structural changes observed by AFM. One would thus expect a different wetting behavior of stearic acid-modified calcite and the surface of an intrinsically hydrophobic material, such as polystyrene. To elucidate this, we followed the change in CA and normalized BD, where the BD at any time, *t*, is normalized by the initial BD, evaluated at *t* = 1.2 s, during droplet evaporation, and quoted as %, that is (BD(*t*)/BD(*t* = 1.2)) × 100 (Figure 6). Note the extended time of these experiments (30 min) compared to those reported in previous sections (30 s). The CA of the water droplet on the polystyrene sample (Figure 6a) decreased slowly with time during the first 5 min, from 98 to 81°. This suggests reorientation of surface groups to expose less hydrophobic sites toward water.^44,45^ The BD was seen to be initially fixed, such that the reduction of the droplet volume due to evaporation was compensated by the decrease in CA. However, after the point where apparently no further rearrangements at the polystyrene surface can occur, the decrease in the CA terminated, and the BD became significantly reduced due to evaporation, that is, at this stage, we encounter the receding CA that is about 80°. About 2 min prior to complete evaporation, the CA decreased again as the droplet contracted to a very small size.

The stearic acid-modified calcite surface is, as discussed above, more complex than polystyrene since in this case, our AFM data show that the stearic acid layer rearranges. Stearic acid can also be partly transferred to the water–air interface of the droplet and to a very limited degree desorbs into the bulk of the droplet. This is reflected in the wetting behavior, as illustrated in Figure 6a,b. We find, thus, that the evaporating droplet also behaves very differently on stearic acid-modified calcite than that on polystyrene. The water CA on stearic acid-modified calcite was found to decrease continuously with time (CA decreased from 107° to about 17° within 17.5 min) as the layer under the droplet became restructured (Figures 3 and 4). In contrast, the BD first increased up to 118% of its initial value during the first 5 min as stearic acid molecules at the interface were removed to the air–water interface, and then, the BD stabilized. This is opposite to the case of the droplet on polystyrene where the CA remained constant, but the BD was shrinking during droplet evaporation. Thus, on stearic acid-modified calcite, the droplet becomes pinned, which is due to the hydrophilic region generated at the droplet edge observed in the AFM image (Figures 3 and 4a) caused by stearic acid desorption at the three phase contact line (TPCL).

#### 4.5.2 Octanoic Acid-Saturated Water

A different behavior was observed for the CA of the octanoic acid-saturated water droplets (Figure 6b). Here, the CA decreased by only 2° during the first 21 min for polystyrene, while the BD was continuously reduced in response to evaporation. About 8 min prior to complete evaporation, the CA started to decrease again as octanoic acid was deposited, and the droplet contracted to very small size.

Just as for polystyrene, the BD of the evaporating C_8_-saturated water droplet was seen to shrink continuously on C_18_-modified calcite. Thus, the presence of C_8_ counteracts removal of stearic acid at the edge of the droplet, and no pinning occurs. The CA was initially stable at around 70°, in accordance with Figure 5. However, after this initial period, the CA decreased continuously with time (CA decreased from 73° to about 32° within 9 min) at the same time as the BD decreased. This is lower than the receding CA, as shown in Table 4. From this, we conclude that C_8_ stabilizes the C_18_ layer for a limited time only. It appears that entropic effects with time lead to some displacement of C_18_ with C_8_ at the calcite surface.

**Table 4:** Advancing and Receding Contact Angle of Milli-Q Water and C_8_-Saturated Milli-Q Water Droplets on Calcite and Carboxylic Acid-Modified Calcite^a^

Droplet	Surface	Advancing CA, θ_a_	Receding CA 1, θ_r_	Receding CA 2, θ_r_	*F*_p_ 1 (mN m^–1^)	*F*_p_ 2 (mN m^–1^)
H_2_O	C_8_-modified	109 ± 3°	41°	15°	4.9	6.8
	C_18_-modified	106 ± 4°	69°	-	2.7	-
C_8_-saturated H_2_O	Freshly fractured calcite	70 ± 1°	58°	47°	0.38	0.35
	C_8_-modified	71 ± 2°	62°	53°	0.28	0.28
	C_18_-modified	74 ± 1°	60°	55°	0.44	0.16

a The advancing CA is an average value obtained during the first 5 s when the contact line starts to advance. Receding CA 1 reflects the CA when the droplet BD starts to decrease, while receding CA 2 is evaluated when the droplet BD stabilizes and remains constant for some time. The resulting pinning force, *F*_p_, values were calculated as: *F*_p_ = γ_LV_(cos θ_a_ – cos θ_r_), where the liquid–vapor surface tension γ_LV_ can be found in Table 2, and θ_a_ and θ_r_ are the advancing and retreating CAs, respectively.

### 4.6 Coffee-Ring Effect

**Figure 7:**
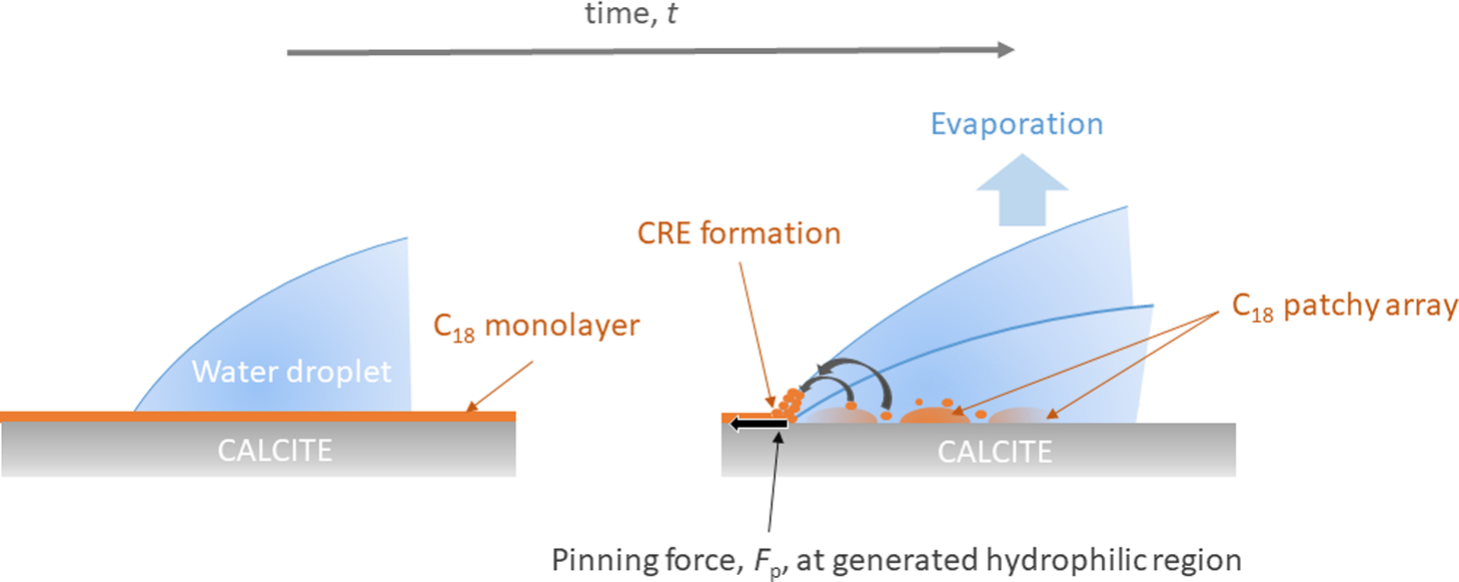
Schematic representation of the rearrangement of the C_18_ stearic acid monolayer on the calcite surface, forming a patchy array of multi-molecular thickness while being in contact with the bulk water droplet, the formation of deposits at the droplet edge due to the CRE during droplet evaporation, and the subsequent pinning of the TPCL.

Evaporation of volatile species combined with pinning of the TPCL results in capillary flow that transports non-volatile solutes to the contact line, giving rise to a CRE. This flow arises from liquid evaporation at the droplet edge that is replenished by liquid flow from the bulk to the edge.^37^ A large CAH appearing as the outcome of droplet pinning at the three-phase contact line can thus result in a CRE during droplet evaporation.^38,46^ In our particular case, this could lead to the transport of carboxylic acids and species dissolved from the calcite surface to the droplet edge. With increasing magnitude of the capillary flow during droplet retraction, the deposition at the contact line has been reported to become more ordered.^47^ The mechanism of this type of CRE development is represented schematically in Figure 7.

For all cases illustrated in Figures 1–4, we note specifically from adhesion and deformation images an accumulation of material at the drop edges. This effect is apparently already for pure water on calcite (Figure 1a), in which case the deposited material is most likely hydrated calcium carbonate. Figure 2a,b shows two cases with C_8_-modified calcite, where exposure to a pure water droplet leads to more accumulation than exposure to a C_8_-saturated aqueous drop. Figure 4a shows the case with pure water on C_18_-modified calcite and the strongest material accumulation at the droplet edge of all cases. In contrast, when the drop is saturated with C_8_ acid (Figure 4b), there is hardly any noticeable effect at the drop edge. A further observation is that results from CA and CAH measurements, as shown in Figures 5 and 6, can be closely related to topographical and nanomechanical images. As one example, Table 4 shows that CA values and CAH are lower for the C_8_ drop on C_18_-modified calcite compared to when pure water is used, which relates to less topographical and nanomechanical effects, as seen in Figure 4b compared to Figure 4a.

We suggest that these observations are typical of the CRE^37,38^ and that it represents a novel case for its occurrence, namely, one containing calcite and fatty acids exposed to a water droplet. The major factor relevant for this type of CRE as a result of hydrophobization is the pinning force estimated from surface tension and CAH.^38^Table 4 shows that the pinning force can be estimated to decrease ten-fold or more for the combinations using C_8_-saturated water compared to pure water on C_8_- or C_18_-modified calcite.

We can clearly suppress the CRE by lowering the pinning force and thereby the capillary flow^37^ by lowering the liquid surface tension in combination with additional C_8_-adsorption that reduces the CAH. The AFM results support these suggestions by demonstrating less material accumulation at the droplet edge, as most apparently seen in the nanomechanical images. Although these experiments are in a model-type situation, we suggest that the findings are of technical importance during carboxylic fatty acid modification of calcium carbonate for the purpose of increased homogeneity in the fatty acid surface layers during deposition and storage of the final product under humid conditions.

## 5 Conclusions

This work has elucidated the stability of bare and fatty carboxylic acid-modified calcite surfaces in the presence of water droplets and droplets of saturated aqueous octanoic acid solutions. AFM was used to follow the structural changes in the unmodified and modified calcite surfaces during short-term (30 s) exposure to the water and saturated octanoic acid droplets. In all cases, more stable surfaces were found in the presence of droplets of saturated aqueous solutions of octanoic acid than that in the presence of pure water droplets. For the bare calcite surface, this is due to adsorption of octanoic acid that retards dissolution and recrystallization of calcite. Adsorption of octanoic acid also occurs on the carboxylic acid-modified surface, and this retards structural rearrangements in the initial monolayer of stearic acid and octanoic acid on the modified calcite surfaces. It also reduces the CAH, which results in smaller pinning force and reduced capillary flow.

The AFM data also allow us to distinguish events occurring at the three-phase surface–liquid–air interface at the water droplet edge and events occurring below the droplet surface. Below the water droplet, the initially hydrophobic stearic acid monolayer is converted into a patchy array in order to reduce the solid–water interfacial energy. We observe clear differences between calcite surfaces modified with octanoic acid and stearic acid. These differences are related to the much higher aqueous solubility of octanoic acid compared to stearic acid. Thus, octanoic acid can dissolve in the bulk of the water droplet, whereas this hardly occurs for stearic acid. However, both types of carboxylic acids can move to the air–water interface, and this results in the creation of a hydrophilic region close to the droplet edge.

The presence of the hydrophilic region at the droplet edge gives rise to significant CAH as the droplet edge becomes pinned, which is the fundamental reason for capillary flow that results in the CRE. The dynamic nature of the carboxylic acid-modified calcite surface results in dramatically different wetting properties during droplet evaporation compared to surfaces of intrinsic hydrophobic materials such as polystyrene where only reorientation of surface groups occurs.

To summarize, this work emphasized the importance of understanding the properties of carboxylic acid layers adsorbed on calcite surfaces with focus on their resistance during exposure to liquid solutions. Such phenomena may arise during industrial processing of modified calcite surfaces and are important to consider during material treatments, storage, and in applications.

## Supporting Information Available

AFM nanomechanical mapping images (40 × 40 μm^2^) of the calcite surface after contact with a water droplet and C_8_-saturated water droplet; AFM nanomechanical mapping images (40 × 40 μm^2^) of the C_8_-modified calcite surface after contact with water and C_8_-saturated water droplets; AFM topography and nanomechanical mapping images (5 × 5 μm^2^) of the C_18_-modified calcite surface after contact with water and C_8_-saturated water droplets prior and after exposure to 25–35 %RH air overnight; and pH as a function of time of solutions containing solid calcite added to water and to saturated aqueous octanoic acid solution (PDF)
