# Supplementary material for: Surface-Modified and Unmodified Calcite: Effects of Water and Saturated Aqueous Octanoic Acid Droplets on Stability and Saturated Fatty Acid Layer Organization
Source: Langmuir. 2021 Nov 18;37(48):14135–46. doi: 10.1021/acs.langmuir.1c02387 (PMC8656169; doi:10.1021/acs.langmuir.1c02387)
Supplement: Supplementary file 1 — la1c02387_si_001.pdf [file la1c02387_si_001.pdf]

Surface modified and unmodified calcite: Effects of water and saturated aqueous octanoic acid droplets on stability and saturated fatty acid layer organization

Natalia A. Wojas [a,b,*], Agne Swerin [c,*], Viveca Wallqvist [a], Mikael Järn [a], Joachim Schoelkopf [d], Patrick A.C. Gane [e], Per M. Claesson [b]

[a] RISE Research Institutes of Sweden, Division of Bioeconomy and Health, Materials and Surface Design Department, Box 5607, SE - 114 86 Stockholm, Sweden

[b] KTH Royal Institute of Technology, School of Engineering Sciences in Chemistry, Biotechnology and Health, Department of Chemistry, Division of Surface and Corrosion Science, Drottning Kristinas väg 51, SE-100 44 Stockholm, Sweden

[c] Karlstad University, Faculty of Health, Science and Technology, Department of Engineering and Chemical Sciences: Chemical Engineering, SE-651 88 Karlstad, Sweden

[d] Omya International AG, Baslerstrasse 42, CH-4665 Oftringen, Switzerland

[e] Aalto University, School of Chemical Engineering, Department of Bioproducts and Biosystems, P.O. Box 16300, FI-00076 Aalto, Finland

* Corresponding authors' emails: natalia.anna.wojas@ri.se and agne.swerin@kau.se

Supporting Information

pH variations

A pH meter (Mettler Toledo) with a small glass membrane holding electrode was used in order to measure the pH in the minimum amount of water (2 – 3 mL) containing small size calcite samples (surface area 120 – 450 mm^2) over 30 min exposure time.

Calcite surface dissolution will affect the solution pH as demonstrated in **Figure S1**. The pH of MilliQ water increases rapidly already within the first 5 s of immersion (see inset) to reach pH 8.2. It further increased to 8.9 after 15 min, and up to 9.2 after 88 h. In the case of a C_8 saturated solution, the pH was initially stable at pH ≈ 4.1 (see inset). However, it increased continuously with time of sample exposure up to 7.4 after 78 h. Large standard deviations especially in the first minutes of exposure are due to variations of the surface area of the calcite added. A pH-gradient in a droplet residing on a calcite surface will induce a flow and could affect the CRE. Based on the data in **Figure S1**, pH-gradients at short times appear to be most likely in water droplets without octanoic acid. If this contributes to the clear CRE seen with water droplets deserves further investigations.

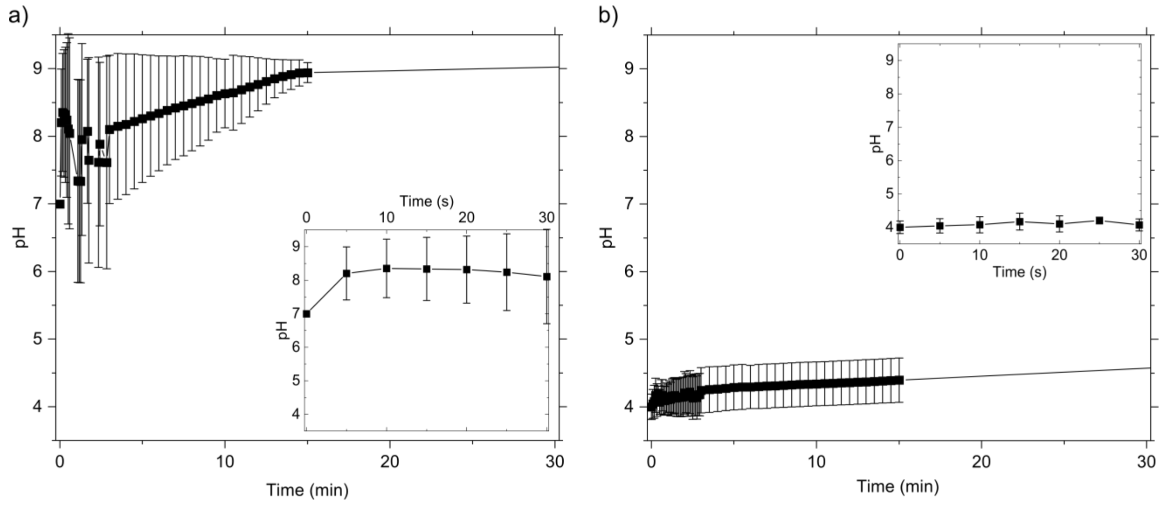

Figure S1. pH as a function of time of solutions containing added solid calcite a) water, and (b) saturated aqueous octanoic acid solution. Insets show pH during the first 30 s.

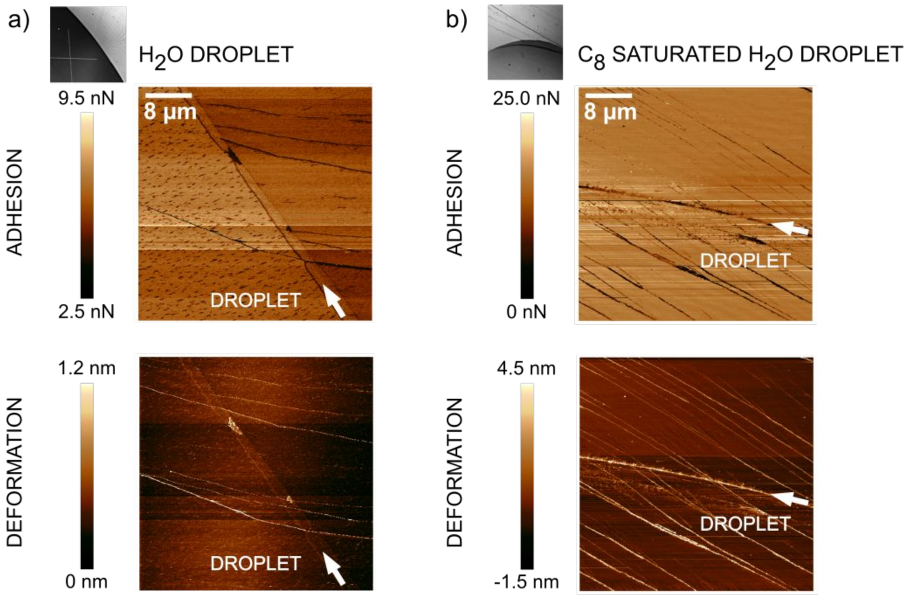

Figure S2. AFM nanomechanical images of the calcite surface after contact with a water droplet (a) and C_8 saturated water droplet (b) directly after exposure to the droplet. The edge of the aqueous droplet is marked by an arrow.

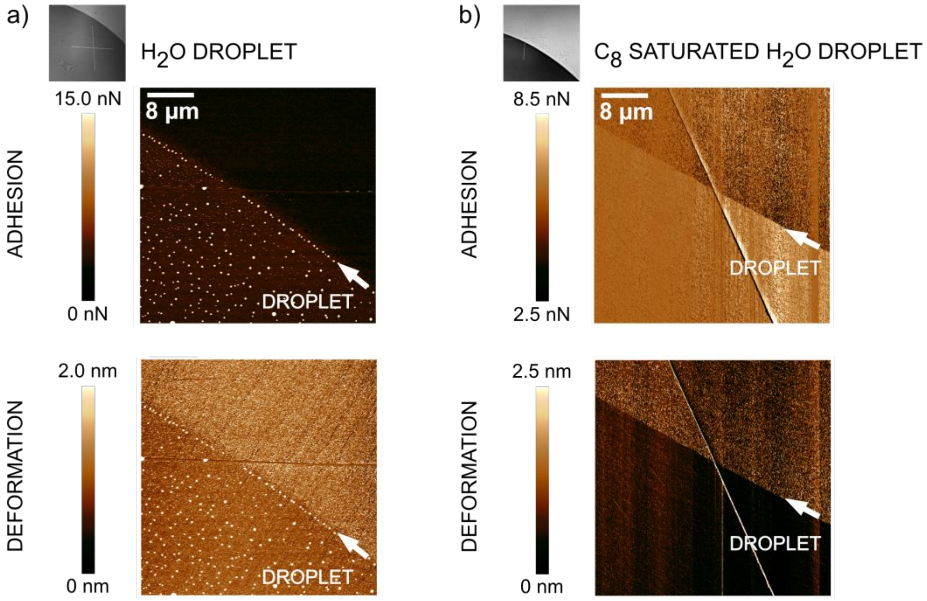

Figure S3. AFM nanomechanical images of the C_8 modified calcite surface after contact with a water (a) and C_8 saturated water droplet (b) directly after exposure to the droplet. The edge of the aqueous droplet is marked by an arrow. The large straight feature seen in panel b is due to the topography and is not induced by the droplet.

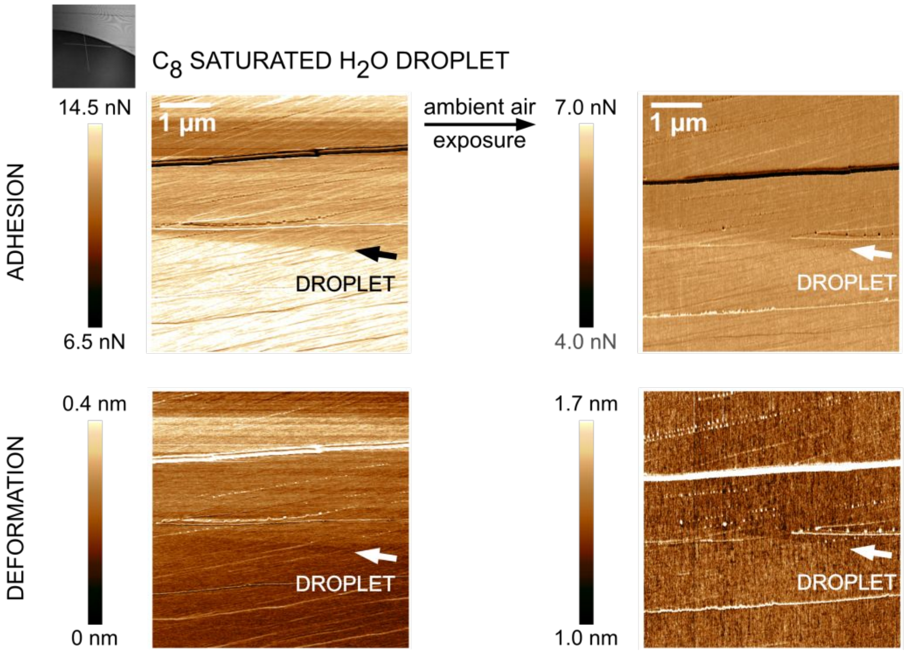

Figure S4. AFM topography and nanomechanical images of the C_{18} modified calcite surface after contact with C_8 saturated water droplet prior and after exposure to 25 - 35 %RH air overnight. The edge of the aqueous droplet is marked by an arrow.
